# Poly[bis­{3,3′-[(biphenyl-4,4′-di­yl)dimethyl­ene]diimidazol-1-ium} γ-octa­molybdate(VI)]

**DOI:** 10.1107/S1600536810010111

**Published:** 2010-03-24

**Authors:** Hongsheng Liu, Lianjiang Su, Limin Wang, Weihong Li

**Affiliations:** a School of Chemistry and Chemical Engineering, Daqing Normal University, Daqing 163712, People’s Republic of China

## Abstract

In the title compound, {(C_20_H_20_N_4_)_2_[Mo_8_O_26_]}_*n*_, the asymmetric unit contains half of an [Mo_8_O_26_]^4−^ anion and one 3,3′-[(biphenyl-4,4′-di­yl)dimethyl­ene]diimidazol-1-ium dication. In the anion, four distorted [MoO_6_] octa­hedra are connected *via* edge-sharing, forming an [Mo_4_O_13_]^2−^ building block, composed of Mo—O(*t*), Mo—O(μ2), Mo—O(μ3) and Mo—O(μ4) units, with Mo—O distances ranging from 1.6858 (15) to 2.4785 (13) Å. The γ-type [Mo_8_O_26_]^4−^ anion is completed by crystallographic inversion symmetry and is linked into an infinite chain along [100] by corner-sharing. The anionic chains and the cations are joined by N—H⋯O hydrogen bonds, generating layers extending parallel to (001).

## Related literature

For backgroud to polyoxomolybdates, see: Zaworotko (1998 ▶); Hong & Do (1998 ▶); Carlucci *et al.* (2003 ▶); Moulton & Zaworotko (2001 ▶). For a similar structure, see: Modec *et al.* (2003 ▶). For the synthesis of 3,3′-(*p*-biphenyl­enedimethyl­ene)diimidazole, see: Fei *et al.* (2000 ▶).
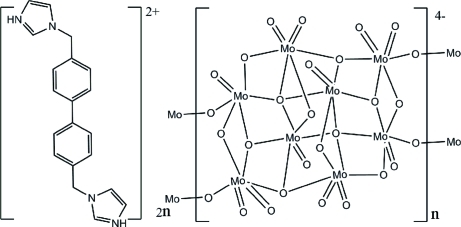


## Experimental

### 

#### Crystal data

(C_20_H_20_N_4_)_2_[Mo_8_O_26_]
*M* *_r_* = 1816.32
Monoclinic, 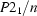

*a* = 9.6460 (4) Å
*b* = 17.3370 (6) Å
*c* = 16.6620 (6) Å
β = 106.145 (1)°
*V* = 2676.54 (17) Å^3^
*Z* = 2
Mo *K*α radiation
μ = 1.90 mm^−1^
*T* = 293 K
0.28 × 0.27 × 0.23 mm

#### Data collection

Bruker APEX CCD area-detector diffractometer
Absorption correction: multi-scan (*SADABS*; Sheldrick, 1996 ▶) *T*_min_ = 0.559, *T*_max_ = 0.616
16209 measured reflections
6405 independent reflections
5716 reflections with *I* > 2σ(*I*)
*R*_int_ = 0.020

#### Refinement

*R*[*F* ^2^ > 2σ(*F* ^2^)] = 0.021
*wR*(*F* ^2^) = 0.051
*S* = 1.03
6405 reflections
378 parameters
2 restraints
H atoms treated by a mixture of independent and constrained refinement
Δρ_max_ = 1.01 e Å^−3^
Δρ_min_ = −0.57 e Å^−3^

### 

Data collection: *SMART* (Bruker, 1997 ▶); cell refinement: *SAINT* (Bruker, 1997) ▶; data reduction: *SAINT*; program(s) used to solve structure: *SHELXS97* (Sheldrick, 2008 ▶); program(s) used to refine structure: *SHELXL97* (Sheldrick, 2008 ▶); molecular graphics: *DIAMOND* (Crystal Impact, 2008 ▶); software used to prepare material for publication: *SHELXL97*.

## Supplementary Material

Crystal structure: contains datablocks global, I. DOI: 10.1107/S1600536810010111/wm2312sup1.cif

Structure factors: contains datablocks I. DOI: 10.1107/S1600536810010111/wm2312Isup2.hkl

Additional supplementary materials:  crystallographic information; 3D view; checkCIF report

## Figures and Tables

**Table 1:** Selected bond lengths (Å)

Mo3—O7	1.6929 (15)
Mo3—O8	1.7365 (14)
Mo3—O6	1.8705 (13)
Mo3—O9	1.9774 (13)
Mo3—O3	2.1723 (14)
Mo3—O9^i^	2.4785 (13)
Mo1—O1	1.6958 (16)
Mo1—O2	1.6996 (15)
Mo1—O13	1.8952 (13)
Mo1—O3	1.9789 (13)
Mo1—O9	2.2882 (14)
Mo1—O8^i^	2.4032 (14)
Mo2—O10	1.6858 (15)
Mo2—O11	1.7665 (15)
Mo2—O12	1.8428 (14)
Mo2—O13	2.0340 (14)
Mo2—O9	2.0869 (12)
Mo2—O6^i^	2.4210 (14)
Mo4—O5	1.6870 (16)
Mo4—O4	1.7163 (16)
Mo4—O12^ii^	1.9289 (14)
Mo4—O3	1.9958 (13)
Mo4—O6	2.1806 (14)
Mo4—O11^i^	2.2857 (15)

Symmetry codes: (i) 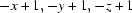; (ii) 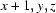.

**Table 2:** Hydrogen-bond geometry (Å, °)

*D*—H⋯*A*	*D*—H	H⋯*A*	*D*⋯*A*	*D*—H⋯*A*
N1—H1*N*⋯O4^iii^	0.92 (3)	1.96 (3)	2.854 (4)	165 (3)
N4—H4*N*⋯O13^iv^	0.92 (3)	1.77 (2)	2.658 (3)	163 (3)

Symmetry codes: (iii) 
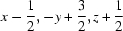
; (iv) 
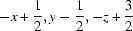
.

**Fig. 1.**
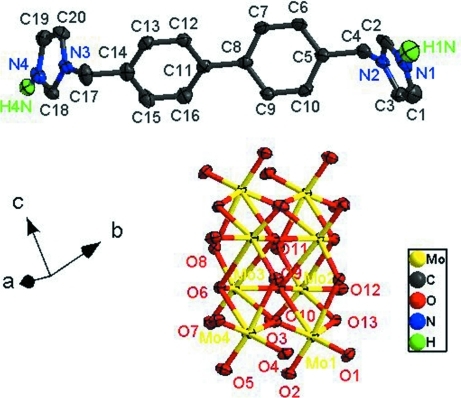
A displacement ellipsoids view of the building units of (I), drawn at the 30% probability level.

**Fig. 2.**
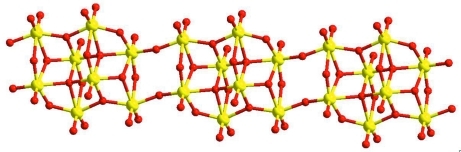
Part of the [Mo8O26]n4n- chain.

**Fig. 3.**
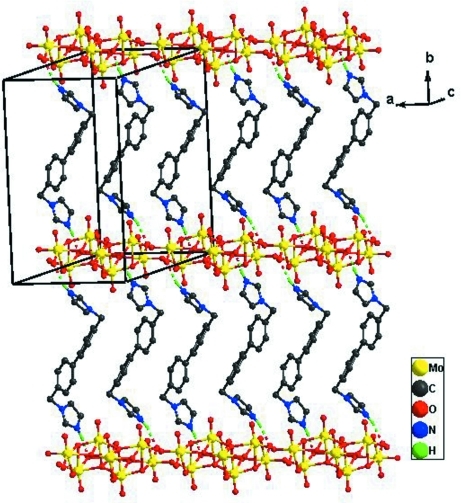
View of the layer structure formed by N-H···O hydrogen bonds.

## supplementary crystallographic information

### Comment

Polyoxometalates of molybdenum are an interesting class of metal-oxido compounds. Recently, many organic-inorganic hybrids have been reported (Zaworotko, 1998; Hong & Do, 1998) because they possess unique architectures and their cooperative functional properties have attracted considerable attention (Carlucci *et al.*, 2003; Moulton & Zaworotko, 2001). In this paper, we present the hydrothermal synthesis and crystal structure of the title compound,  (C_20_H_20_N_4_)_2_[Mo_8_O_26_], (I), based on the protonated 3,3'-(p-biphenylenedimethylene)diimidazole ligand (hereafter *L*), and a γ-type [Mo_8_O_26_]^4-^ POM anion.

In the structure of compound (I), the asymmetric unit contains half of a γ-type [Mo_8_O_26_]^4-^ anion, which is completed by inversion symmetry, and one H_2_*L*^2+^ cation (Fig. 1). The four unique [MoO_6_] octahedra are considerably distorted. They are connected via edge-sharing to form the [Mo_4_O_13_]^2-^ unit, which is further linked together by edge-sharing to give rise to γ-[Mo_8_O_26_]^4-^ octamolybdate units. Four sets of Mo-O distances, corresponding to their function as Mo-O(t), Mo-O(µ2), Mo-O(µ3) and Mo-O(µ4) units, are observed and range from 1.6858 (15) Å to 2.4785 (13) Å. These distances are similar to those in comparable structures (Modec *et al.*, 2003). The γ-octamolybdate units are finally linked together through sharing common vertices to form infinite chains extending along [100] (Fig. 2). These chains are linked through N—H···O hydrogen bonding to generate layers extending parallel to (001) (Fig. 3).

### Experimental

A mixture of (NH_4_)_6_Mo_7_O_24_^.^4H_2_O (0.12 g, 0.1 mmol), *L* (0.031 g, 0.2 mmol) (Fei *et al.*, 2000) and H_2_O (10 ml) was adjusted with HCl (6M) to pH = 4-5. Then the mixture was placed in a 23 ml Teflon-lined stainless steel container. The container was heated to 423 K and held at that temperature for 72 h, and cooled to room temperature. Colorless crystals were collected in 67% yield.

### Refinement

All H atoms on C atoms were positioned geometrically and refined as riding atoms, with C—H = 0.93 Å for aromatic C atoms and C—H = 0.97 Å for aliphatic C atoms, and *U*_iso_(H) = 1.2*U*_eq_ and 1.5*U*_eq_(C), respectively. H atoms of the protonated N atoms in the cation were located in a difference Fourier map and were refined freely.

### Crystal data

**Table:**

(C_20_H_20_N_4_)_2_[Mo_8_O_26_]	*F*(000) = 1760
*M**_r_* = 1816.32	*D*_x_ = 2.254 Mg m^−^^3^
Monoclinic, *P*2_1_/*n*	Mo *K*α radiation, λ = 0.71069 Å
Hall symbol: -P 2yn	Cell parameters from 139 reflections
*a* = 9.6460 (4) Å	θ = 1.7–28.3°
*b* = 17.3370 (6) Å	µ = 1.90 mm^−^^1^
*c* = 16.6620 (6) Å	*T* = 293 K
β = 106.145 (1)°	Block, colorless
*V* = 2676.54 (17) Å^3^	0.28 × 0.27 × 0.23 mm
*Z* = 2	

### Data collection

**Table:**

Bruker APEX CCD area-detector diffractometer	6405 independent reflections
Radiation source: fine-focus sealed tube	5716 reflections with *I* > 2σ(*I*)
graphite	*R*_int_ = 0.020
ω scans	θ_max_ = 28.3°, θ_min_ = 1.7°
Absorption correction: multi-scan (*SADABS*; Sheldrick, 1996)	*h* = −9→12
*T*_min_ = 0.559, *T*_max_ = 0.616	*k* = −23→23
16209 measured reflections	*l* = −21→21

### Refinement

**Table:**

Refinement on *F*^2^	Primary atom site location: structure-invariant direct methods
Least-squares matrix: full	Secondary atom site location: difference Fourier map
*R*[*F*^2^ > 2σ(*F*^2^)] = 0.021	Hydrogen site location: inferred from neighbouring sites
*wR*(*F*^2^) = 0.051	H atoms treated by a mixture of independent and constrained refinement
*S* = 1.03	*w* = 1/[σ^2^(*F*_o_^2^) + (0.0254*P*)^2^ + 0.027*P*] where *P* = (*F*_o_^2^ + 2*F*_c_^2^)/3
6405 reflections	(Δ/σ)_max_ = 0.001
378 parameters	Δρ_max_ = 1.01 e Å^−^^3^
2 restraints	Δρ_min_ = −0.57 e Å^−^^3^

### Special details

**Table:**

Geometry. All esds (except the esd in the dihedral angle between two l.s. planes) are estimated using the full covariance matrix. The cell esds are taken into account individually in the estimation of esds in distances, angles and torsion angles; correlations between esds in cell parameters are only used when they are defined by crystal symmetry. An approximate (isotropic) treatment of cell esds is used for estimating esds involving l.s. planes.
Refinement. Refinement of F^2^ against ALL reflections. The weighted R-factor wR and goodness of fit S are based on F^2^, conventional R-factors R are based on F, with F set to zero for negative F^2^. The threshold expression of F^2^ > 2sigma(F^2^) is used only for calculating R-factors(gt) etc. and is not relevant to the choice of reflections for refinement. R-factors based on F^2^ are statistically about twice as large as those based on F, and R- factors based on ALL data will be even larger.

### Fractional atomic coordinates and isotropic or equivalent isotropic displacement parameters (Å^2^)

**Table:**

	*x*	*y*	*z*	*U*_iso_*/*U*_eq_	
Mo3	0.589310 (17)	0.411089 (9)	0.498376 (10)	0.02082 (5)	
Mo1	0.363467 (17)	0.472925 (10)	0.320138 (10)	0.02348 (5)	
Mo2	0.172190 (17)	0.433192 (10)	0.449319 (11)	0.02298 (5)	
Mo4	0.773555 (17)	0.457531 (10)	0.370756 (11)	0.02573 (5)	
C8	0.1996 (3)	0.51860 (14)	0.97308 (16)	0.0430 (6)	
O11	0.20748 (15)	0.42795 (8)	0.55905 (9)	0.0297 (3)	
O7	0.56742 (16)	0.31831 (8)	0.46558 (10)	0.0361 (4)	
O8	0.60243 (15)	0.40726 (8)	0.60437 (9)	0.0287 (3)	
C14	0.4967 (3)	0.33842 (16)	1.08281 (18)	0.0495 (7)	
O9	0.39012 (13)	0.45144 (8)	0.45911 (8)	0.0226 (3)	
O2	0.34754 (16)	0.38200 (9)	0.28046 (10)	0.0388 (4)	
O4	0.74877 (17)	0.51414 (10)	0.28339 (10)	0.0410 (4)	
C7	0.1760 (3)	0.57956 (18)	1.02188 (17)	0.0569 (8)	
H7	0.2222	0.5799	1.0789	0.068*	
O10	0.17148 (18)	0.33876 (9)	0.42508 (11)	0.0420 (4)	
O5	0.76131 (17)	0.36631 (10)	0.33422 (11)	0.0441 (4)	
C17	0.6025 (3)	0.27601 (17)	1.1218 (2)	0.0643 (9)	
H17A	0.6299	0.2481	1.0781	0.077*	
H17B	0.6888	0.2995	1.1578	0.077*	
O6	0.77906 (14)	0.43579 (8)	0.50051 (9)	0.0251 (3)	
C11	0.3006 (3)	0.45522 (15)	1.01032 (16)	0.0420 (6)	
O12	−0.01976 (15)	0.46101 (10)	0.41738 (10)	0.0375 (4)	
O13	0.18052 (14)	0.48333 (8)	0.34039 (9)	0.0264 (3)	
O3	0.57176 (14)	0.46661 (8)	0.37908 (9)	0.0253 (3)	
N2	−0.0262 (2)	0.75613 (12)	0.81224 (14)	0.0502 (6)	
C19	0.4540 (3)	0.17163 (17)	1.2659 (2)	0.0614 (8)	
H19	0.4247	0.1639	1.3139	0.074*	
C18	0.4929 (3)	0.15197 (14)	1.14441 (19)	0.0488 (7)	
H18	0.4954	0.1293	1.0942	0.059*	
C12	0.4210 (3)	0.46984 (15)	1.07717 (16)	0.0471 (6)	
H12	0.4371	0.5196	1.0985	0.057*	
N3	0.5424 (2)	0.22120 (11)	1.17101 (14)	0.0443 (5)	
C10	0.0366 (3)	0.57963 (16)	0.85391 (17)	0.0501 (7)	
H10	−0.0108	0.5790	0.7971	0.060*	
C20	0.5186 (3)	0.23459 (16)	1.24635 (19)	0.0596 (8)	
H20	0.5426	0.2791	1.2784	0.071*	
C6	0.0857 (3)	0.63925 (18)	0.98732 (18)	0.0605 (8)	
H6	0.0726	0.6796	1.0213	0.073*	
N1	0.1007 (3)	0.84206 (16)	0.7726 (3)	0.0811 (9)	
C16	0.2814 (3)	0.37990 (17)	0.9806 (2)	0.0592 (7)	
H16	0.2019	0.3682	0.9360	0.071*	
C2	0.0738 (3)	0.81019 (17)	0.8382 (2)	0.0602 (8)	
H2	0.1175	0.8233	0.8936	0.072*	
C4	−0.0864 (3)	0.70583 (17)	0.86535 (18)	0.0559 (7)	
H4A	−0.1776	0.6847	0.8322	0.067*	
H4B	−0.1054	0.7363	0.9100	0.067*	
C15	0.3770 (3)	0.32231 (17)	1.0156 (2)	0.0640 (8)	
H15	0.3619	0.2724	0.9944	0.077*	
N4	0.4398 (2)	0.12085 (13)	1.20108 (16)	0.0506 (6)	
C3	−0.0639 (4)	0.7547 (2)	0.7282 (2)	0.0794 (10)	
H3	−0.1323	0.7225	0.6938	0.095*	
C5	0.0136 (3)	0.64072 (15)	0.90269 (17)	0.0473 (6)	
O1	0.35190 (17)	0.53203 (10)	0.23743 (10)	0.0406 (4)	
C13	0.5162 (3)	0.41255 (16)	1.11222 (17)	0.0496 (6)	
H13	0.5957	0.4242	1.1569	0.059*	
C9	0.1282 (3)	0.52027 (15)	0.88812 (17)	0.0493 (6)	
H9	0.1428	0.4805	0.8539	0.059*	
C1	0.0152 (5)	0.8082 (2)	0.7031 (3)	0.0932 (12)	
H1	0.0120	0.8199	0.6482	0.112*	
H4N	0.396 (3)	0.0737 (9)	1.1971 (17)	0.057 (8)*	
H1N	0.163 (3)	0.8830 (14)	0.777 (2)	0.102 (14)*	

### Atomic displacement parameters (Å^2^)

**Table:**

	*U*^11^	*U*^22^	*U*^33^	*U*^12^	*U*^13^	*U*^23^
Mo3	0.01444 (8)	0.02311 (9)	0.02444 (10)	−0.00012 (6)	0.00463 (6)	0.00029 (7)
Mo1	0.01472 (8)	0.03481 (10)	0.02030 (9)	−0.00007 (7)	0.00384 (6)	−0.00334 (7)
Mo2	0.01459 (8)	0.02982 (9)	0.02530 (10)	−0.00497 (6)	0.00681 (7)	−0.00595 (7)
Mo4	0.01425 (8)	0.03941 (10)	0.02425 (10)	−0.00371 (7)	0.00657 (7)	−0.00845 (8)
C8	0.0415 (14)	0.0504 (14)	0.0407 (14)	−0.0023 (11)	0.0170 (11)	−0.0041 (12)
O11	0.0254 (8)	0.0355 (8)	0.0298 (8)	−0.0068 (6)	0.0105 (6)	−0.0017 (6)
O7	0.0346 (9)	0.0276 (7)	0.0442 (10)	−0.0007 (7)	0.0079 (7)	−0.0045 (7)
O8	0.0253 (7)	0.0331 (8)	0.0274 (8)	−0.0014 (6)	0.0068 (6)	0.0033 (6)
C14	0.0369 (14)	0.0525 (16)	0.0620 (18)	−0.0001 (12)	0.0186 (13)	0.0083 (14)
O9	0.0146 (6)	0.0288 (7)	0.0237 (7)	−0.0016 (5)	0.0045 (5)	−0.0018 (6)
O2	0.0276 (8)	0.0468 (9)	0.0410 (10)	−0.0002 (7)	0.0080 (7)	−0.0174 (8)
O4	0.0357 (9)	0.0605 (11)	0.0289 (9)	−0.0051 (8)	0.0127 (7)	−0.0036 (8)
C7	0.0601 (19)	0.075 (2)	0.0342 (15)	0.0158 (15)	0.0107 (13)	−0.0071 (14)
O10	0.0454 (10)	0.0343 (8)	0.0469 (11)	−0.0085 (8)	0.0140 (8)	−0.0126 (8)
O5	0.0322 (9)	0.0491 (10)	0.0513 (11)	−0.0022 (7)	0.0119 (8)	−0.0216 (8)
C17	0.0440 (17)	0.0597 (18)	0.092 (3)	0.0050 (14)	0.0239 (16)	0.0152 (17)
O6	0.0139 (6)	0.0326 (7)	0.0282 (8)	−0.0001 (5)	0.0047 (5)	−0.0022 (6)
C11	0.0411 (14)	0.0490 (14)	0.0395 (14)	−0.0016 (11)	0.0175 (11)	0.0023 (11)
O12	0.0154 (7)	0.0637 (11)	0.0346 (9)	−0.0039 (7)	0.0088 (6)	−0.0116 (8)
O13	0.0152 (6)	0.0377 (8)	0.0248 (8)	0.0013 (6)	0.0034 (5)	−0.0035 (6)
O3	0.0149 (7)	0.0379 (8)	0.0235 (7)	−0.0012 (6)	0.0061 (5)	−0.0010 (6)
N2	0.0476 (13)	0.0443 (12)	0.0503 (14)	0.0066 (10)	−0.0002 (10)	−0.0195 (11)
C19	0.068 (2)	0.0582 (18)	0.059 (2)	0.0014 (16)	0.0190 (16)	0.0012 (15)
C18	0.0382 (14)	0.0418 (14)	0.0654 (19)	0.0008 (11)	0.0128 (13)	−0.0155 (13)
C12	0.0548 (17)	0.0480 (15)	0.0405 (15)	−0.0017 (13)	0.0166 (13)	−0.0030 (12)
N3	0.0352 (11)	0.0356 (10)	0.0589 (14)	0.0023 (9)	0.0079 (10)	−0.0045 (10)
C10	0.0451 (16)	0.0588 (17)	0.0380 (15)	−0.0015 (13)	−0.0023 (11)	−0.0092 (12)
C20	0.072 (2)	0.0412 (14)	0.062 (2)	−0.0015 (14)	0.0122 (16)	−0.0134 (14)
C6	0.065 (2)	0.071 (2)	0.0432 (17)	0.0190 (16)	0.0125 (14)	−0.0144 (15)
N1	0.066 (2)	0.0483 (16)	0.135 (3)	0.0127 (15)	0.039 (2)	−0.0014 (19)
C16	0.0421 (16)	0.0581 (18)	0.069 (2)	−0.0050 (13)	0.0012 (13)	−0.0070 (15)
C2	0.0354 (15)	0.0547 (17)	0.085 (2)	0.0081 (13)	0.0071 (15)	−0.0322 (17)
C4	0.0434 (16)	0.0637 (18)	0.0559 (18)	0.0078 (13)	0.0058 (13)	−0.0137 (15)
C15	0.0540 (19)	0.0477 (16)	0.084 (2)	−0.0041 (14)	0.0095 (16)	−0.0063 (16)
N4	0.0356 (12)	0.0392 (12)	0.0733 (17)	−0.0051 (10)	0.0089 (11)	−0.0091 (12)
C3	0.116 (3)	0.059 (2)	0.051 (2)	0.000 (2)	0.0034 (19)	−0.0105 (17)
C5	0.0365 (14)	0.0552 (16)	0.0483 (16)	0.0029 (12)	0.0084 (11)	−0.0095 (13)
O1	0.0363 (9)	0.0583 (11)	0.0261 (9)	0.0010 (8)	0.0067 (7)	0.0061 (8)
C13	0.0428 (15)	0.0604 (17)	0.0438 (16)	−0.0044 (13)	0.0091 (12)	0.0008 (13)
C9	0.0518 (16)	0.0482 (15)	0.0462 (16)	−0.0018 (12)	0.0107 (13)	−0.0136 (13)
C1	0.129 (4)	0.078 (3)	0.072 (3)	0.014 (3)	0.027 (3)	0.007 (2)

### Geometric parameters (Å, °)

**Table:**

Mo3—O7	1.6929 (15)	O6—Mo2^i^	2.4210 (14)
Mo3—O8	1.7365 (14)	C11—C12	1.390 (4)
Mo3—O6	1.8705 (13)	C11—C16	1.391 (4)
Mo3—O9	1.9774 (13)	O12—Mo4^iii^	1.9289 (14)
Mo3—O3	2.1723 (14)	N2—C2	1.328 (3)
Mo3—O9^i^	2.4785 (13)	N2—C3	1.346 (4)
Mo1—O1	1.6958 (16)	N2—C4	1.472 (4)
Mo1—O2	1.6996 (15)	C19—C20	1.341 (4)
Mo1—O13	1.8952 (13)	C19—N4	1.370 (4)
Mo1—O3	1.9789 (13)	C19—H19	0.9300
Mo1—O9	2.2882 (14)	C18—N4	1.309 (4)
Mo1—O8^i^	2.4032 (14)	C18—N3	1.322 (3)
Mo2—O10	1.6858 (15)	C18—H18	0.9300
Mo2—O11	1.7665 (15)	C12—C13	1.369 (4)
Mo2—O12	1.8428 (14)	C12—H12	0.9300
Mo2—O13	2.0340 (14)	N3—C20	1.357 (3)
Mo2—O9	2.0869 (12)	C10—C9	1.373 (4)
Mo2—O6^i^	2.4210 (14)	C10—C5	1.390 (4)
Mo4—O5	1.6870 (16)	C10—H10	0.9300
Mo4—O4	1.7163 (16)	C20—H20	0.9300
Mo4—O12^ii^	1.9289 (14)	C6—C5	1.388 (4)
Mo4—O3	1.9958 (13)	C6—H6	0.9300
Mo4—O6	2.1806 (14)	N1—C2	1.314 (4)
Mo4—O11^i^	2.2857 (15)	N1—C1	1.354 (5)
C8—C7	1.390 (3)	N1—H1N	0.92 (3)
C8—C9	1.392 (4)	C16—C15	1.374 (4)
C8—C11	1.485 (4)	C16—H16	0.9300
O11—Mo4^i^	2.2857 (15)	C2—H2	0.9300
O8—Mo1^i^	2.4032 (14)	C4—C5	1.503 (4)
C14—C13	1.370 (4)	C4—H4A	0.9700
C14—C15	1.396 (4)	C4—H4B	0.9700
C14—C17	1.505 (4)	C15—H15	0.9300
O9—Mo3^i^	2.4785 (13)	N4—H4N	0.92 (3)
C7—C6	1.372 (4)	C3—C1	1.339 (5)
C7—H7	0.9300	C3—H3	0.9300
C17—N3	1.475 (3)	C13—H13	0.9300
C17—H17A	0.9700	C9—H9	0.9300
C17—H17B	0.9700	C1—H1	0.9300
			
O7—Mo3—O8	105.02 (7)	N3—C17—C14	112.3 (2)
O7—Mo3—O6	104.90 (7)	N3—C17—H17A	109.1
O8—Mo3—O6	101.26 (6)	C14—C17—H17A	109.1
O7—Mo3—O9	101.94 (7)	N3—C17—H17B	109.1
O8—Mo3—O9	98.01 (6)	C14—C17—H17B	109.1
O6—Mo3—O9	141.35 (6)	H17A—C17—H17B	107.9
O7—Mo3—O3	98.90 (7)	Mo3—O6—Mo4	105.48 (6)
O8—Mo3—O3	155.88 (6)	Mo3—O6—Mo2^i^	108.45 (6)
O6—Mo3—O3	75.10 (6)	Mo4—O6—Mo2^i^	97.41 (5)
O9—Mo3—O3	73.69 (5)	C12—C11—C16	117.1 (3)
O7—Mo3—O9^i^	177.09 (6)	C12—C11—C8	120.3 (2)
O8—Mo3—O9^i^	76.63 (6)	C16—C11—C8	122.6 (3)
O6—Mo3—O9^i^	76.95 (5)	Mo2—O12—Mo4^iii^	162.00 (10)
O9—Mo3—O9^i^	75.38 (5)	Mo1—O13—Mo2	112.83 (7)
O3—Mo3—O9^i^	79.34 (5)	Mo1—O3—Mo4	147.69 (8)
O1—Mo1—O2	105.42 (8)	Mo1—O3—Mo3	106.78 (6)
O1—Mo1—O13	103.48 (7)	Mo4—O3—Mo3	101.50 (6)
O2—Mo1—O13	99.76 (6)	C2—N2—C3	108.3 (3)
O1—Mo1—O3	105.37 (7)	C2—N2—C4	126.5 (3)
O2—Mo1—O3	96.38 (7)	C3—N2—C4	125.3 (3)
O13—Mo1—O3	141.63 (6)	C20—C19—N4	106.5 (3)
O1—Mo1—O9	152.15 (7)	C20—C19—H19	126.8
O2—Mo1—O9	102.43 (7)	N4—C19—H19	126.8
O13—Mo1—O9	71.54 (5)	N4—C18—N3	108.0 (2)
O3—Mo1—O9	71.09 (5)	N4—C18—H18	126.0
O1—Mo1—O8^i^	82.62 (7)	N3—C18—H18	126.0
O2—Mo1—O8^i^	171.74 (7)	C13—C12—C11	121.4 (2)
O13—Mo1—O8^i^	79.82 (5)	C13—C12—H12	119.3
O3—Mo1—O8^i^	79.38 (5)	C11—C12—H12	119.3
O9—Mo1—O8^i^	69.54 (5)	C18—N3—C20	109.1 (2)
O10—Mo2—O11	100.65 (8)	C18—N3—C17	124.0 (3)
O10—Mo2—O12	104.37 (8)	C20—N3—C17	126.7 (2)
O11—Mo2—O12	101.62 (7)	C9—C10—C5	121.2 (2)
O10—Mo2—O13	101.52 (7)	C9—C10—H10	119.4
O11—Mo2—O13	154.35 (6)	C5—C10—H10	119.4
O12—Mo2—O13	85.20 (6)	C19—C20—N3	107.1 (3)
O10—Mo2—O9	95.90 (7)	C19—C20—H20	126.4
O11—Mo2—O9	91.54 (6)	N3—C20—H20	126.4
O12—Mo2—O9	153.16 (7)	C7—C6—C5	121.4 (3)
O13—Mo2—O9	73.51 (5)	C7—C6—H6	119.3
O10—Mo2—O6^i^	168.92 (7)	C5—C6—H6	119.3
O11—Mo2—O6^i^	74.21 (6)	C2—N1—C1	108.3 (3)
O12—Mo2—O6^i^	86.40 (6)	C2—N1—H1N	123 (3)
O13—Mo2—O6^i^	81.67 (5)	C1—N1—H1N	129 (3)
O9—Mo2—O6^i^	74.69 (5)	C15—C16—C11	121.6 (3)
O5—Mo4—O4	104.52 (8)	C15—C16—H16	119.2
O5—Mo4—O12^ii^	97.79 (7)	C11—C16—H16	119.2
O4—Mo4—O12^ii^	102.02 (7)	N1—C2—N2	108.6 (3)
O5—Mo4—O3	97.31 (7)	N1—C2—H2	125.7
O4—Mo4—O3	96.05 (7)	N2—C2—H2	125.7
O12^ii^—Mo4—O3	152.67 (6)	N2—C4—C5	112.3 (2)
O5—Mo4—O6	100.12 (7)	N2—C4—H4A	109.2
O4—Mo4—O6	154.02 (7)	C5—C4—H4A	109.2
O12^ii^—Mo4—O6	82.45 (6)	N2—C4—H4B	109.2
O3—Mo4—O6	72.55 (5)	C5—C4—H4B	109.2
O5—Mo4—O11^i^	170.62 (7)	H4A—C4—H4B	107.9
O4—Mo4—O11^i^	84.82 (7)	C16—C15—C14	120.2 (3)
O12^ii^—Mo4—O11^i^	80.80 (6)	C16—C15—H15	119.9
O3—Mo4—O11^i^	80.61 (5)	C14—C15—H15	119.9
O6—Mo4—O11^i^	70.51 (5)	C18—N4—C19	109.3 (2)
C7—C8—C9	117.6 (2)	C18—N4—H4N	125.6 (17)
C7—C8—C11	120.9 (2)	C19—N4—H4N	125.0 (17)
C9—C8—C11	121.5 (2)	C1—C3—N2	107.4 (3)
Mo2—O11—Mo4^i^	116.73 (7)	C1—C3—H3	126.3
Mo3—O8—Mo1^i^	117.38 (7)	N2—C3—H3	126.3
C13—C14—C15	118.4 (3)	C6—C5—C10	117.6 (3)
C13—C14—C17	120.7 (3)	C6—C5—C4	121.1 (2)
C15—C14—C17	120.9 (3)	C10—C5—C4	121.3 (2)
Mo3—O9—Mo2	146.85 (7)	C12—C13—C14	121.3 (3)
Mo3—O9—Mo1	102.58 (5)	C12—C13—H13	119.4
Mo2—O9—Mo1	96.79 (5)	C14—C13—H13	119.4
Mo3—O9—Mo3^i^	104.62 (5)	C10—C9—C8	121.2 (2)
Mo2—O9—Mo3^i^	99.61 (5)	C10—C9—H9	119.4
Mo1—O9—Mo3^i^	96.33 (5)	C8—C9—H9	119.4
C6—C7—C8	121.1 (3)	C3—C1—N1	107.4 (4)
C6—C7—H7	119.5	C3—C1—H1	126.3
C8—C7—H7	119.5	N1—C1—H1	126.3
			
O10—Mo2—O11—Mo4^i^	178.62 (8)	O10—Mo2—O12—Mo4^iii^	−5.7 (3)
O12—Mo2—O11—Mo4^i^	−74.14 (8)	O11—Mo2—O12—Mo4^iii^	−110.1 (3)
O13—Mo2—O11—Mo4^i^	29.20 (17)	O13—Mo2—O12—Mo4^iii^	94.9 (3)
O9—Mo2—O11—Mo4^i^	82.32 (7)	O9—Mo2—O12—Mo4^iii^	132.1 (3)
O6^i^—Mo2—O11—Mo4^i^	8.70 (5)	O6^i^—Mo2—O12—Mo4^iii^	176.9 (3)
O7—Mo3—O8—Mo1^i^	179.62 (7)	O1—Mo1—O13—Mo2	−170.08 (8)
O6—Mo3—O8—Mo1^i^	70.66 (8)	O2—Mo1—O13—Mo2	81.34 (8)
O9—Mo3—O8—Mo1^i^	−75.66 (7)	O3—Mo1—O13—Mo2	−32.19 (13)
O3—Mo3—O8—Mo1^i^	−7.98 (18)	O9—Mo1—O13—Mo2	−18.61 (6)
O9^i^—Mo3—O8—Mo1^i^	−2.85 (6)	O8^i^—Mo1—O13—Mo2	−90.29 (7)
O7—Mo3—O9—Mo2	45.50 (14)	O10—Mo2—O13—Mo1	−72.56 (9)
O8—Mo3—O9—Mo2	−61.79 (14)	O11—Mo2—O13—Mo1	76.76 (15)
O6—Mo3—O9—Mo2	178.76 (10)	O12—Mo2—O13—Mo1	−176.27 (8)
O3—Mo3—O9—Mo2	141.41 (14)	O9—Mo2—O13—Mo1	20.25 (6)
O9^i^—Mo3—O9—Mo2	−135.65 (16)	O6^i^—Mo2—O13—Mo1	96.67 (7)
O7—Mo3—O9—Mo1	−78.78 (7)	O1—Mo1—O3—Mo4	−41.21 (16)
O8—Mo3—O9—Mo1	173.93 (6)	O2—Mo1—O3—Mo4	66.75 (15)
O6—Mo3—O9—Mo1	54.48 (11)	O13—Mo1—O3—Mo4	−178.65 (11)
O3—Mo3—O9—Mo1	17.13 (5)	O9—Mo1—O3—Mo4	167.74 (16)
O9^i^—Mo3—O9—Mo1	100.07 (6)	O8^i^—Mo1—O3—Mo4	−120.42 (15)
O7—Mo3—O9—Mo3^i^	−178.85 (6)	O1—Mo1—O3—Mo3	168.78 (7)
O8—Mo3—O9—Mo3^i^	73.85 (6)	O2—Mo1—O3—Mo3	−83.27 (8)
O6—Mo3—O9—Mo3^i^	−45.59 (11)	O13—Mo1—O3—Mo3	31.33 (12)
O3—Mo3—O9—Mo3^i^	−82.95 (6)	O9—Mo1—O3—Mo3	17.72 (5)
O9^i^—Mo3—O9—Mo3^i^	0.0	O8^i^—Mo1—O3—Mo3	89.56 (6)
O10—Mo2—O9—Mo3	−40.83 (14)	O5—Mo4—O3—Mo1	−68.50 (15)
O11—Mo2—O9—Mo3	60.04 (13)	O4—Mo4—O3—Mo1	37.03 (15)
O12—Mo2—O9—Mo3	179.98 (11)	O12^ii^—Mo4—O3—Mo1	168.42 (12)
O13—Mo2—O9—Mo3	−141.13 (14)	O6—Mo4—O3—Mo1	−166.86 (15)
O6^i^—Mo2—O9—Mo3	133.21 (14)	O11^i^—Mo4—O3—Mo1	120.74 (15)
O10—Mo2—O9—Mo1	84.87 (7)	O5—Mo4—O3—Mo3	82.27 (8)
O11—Mo2—O9—Mo1	−174.26 (6)	O4—Mo4—O3—Mo3	−172.19 (7)
O12—Mo2—O9—Mo1	−54.32 (15)	O12^ii^—Mo4—O3—Mo3	−40.81 (17)
O13—Mo2—O9—Mo1	−15.43 (5)	O6—Mo4—O3—Mo3	−16.09 (5)
O6^i^—Mo2—O9—Mo1	−101.10 (6)	O11^i^—Mo4—O3—Mo3	−88.48 (6)
O10—Mo2—O9—Mo3^i^	−177.51 (7)	O7—Mo3—O3—Mo1	79.61 (7)
O11—Mo2—O9—Mo3^i^	−76.64 (6)	O8—Mo3—O3—Mo1	−92.96 (14)
O12—Mo2—O9—Mo3^i^	43.30 (15)	O6—Mo3—O3—Mo1	−177.22 (7)
O13—Mo2—O9—Mo3^i^	82.19 (6)	O9—Mo3—O3—Mo1	−20.31 (6)
O6^i^—Mo2—O9—Mo3^i^	−3.47 (4)	O9^i^—Mo3—O3—Mo1	−98.04 (6)
O1—Mo1—O9—Mo3	−106.35 (13)	O7—Mo3—O3—Mo4	−84.57 (7)
O2—Mo1—O9—Mo3	73.42 (7)	O8—Mo3—O3—Mo4	102.86 (14)
O13—Mo1—O9—Mo3	169.71 (7)	O6—Mo3—O3—Mo4	18.60 (6)
O3—Mo1—O9—Mo3	−19.15 (6)	O9—Mo3—O3—Mo4	175.51 (7)
O8^i^—Mo1—O9—Mo3	−104.55 (6)	O9^i^—Mo3—O3—Mo4	97.78 (6)
O1—Mo1—O9—Mo2	100.72 (13)	C16—C11—C12—C13	−0.1 (4)
O2—Mo1—O9—Mo2	−79.51 (7)	C8—C11—C12—C13	−179.6 (2)
O13—Mo1—O9—Mo2	16.78 (5)	N4—C18—N3—C20	−0.6 (3)
O3—Mo1—O9—Mo2	−172.08 (7)	N4—C18—N3—C17	−176.1 (2)
O8^i^—Mo1—O9—Mo2	102.52 (6)	C14—C17—N3—C18	101.9 (3)
O1—Mo1—O9—Mo3^i^	0.21 (15)	C14—C17—N3—C20	−72.8 (4)
O2—Mo1—O9—Mo3^i^	179.98 (6)	N4—C19—C20—N3	−0.1 (3)
O13—Mo1—O9—Mo3^i^	−83.73 (6)	C18—N3—C20—C19	0.4 (3)
O3—Mo1—O9—Mo3^i^	87.41 (5)	C17—N3—C20—C19	175.7 (3)
O8^i^—Mo1—O9—Mo3^i^	2.01 (4)	C8—C7—C6—C5	0.6 (5)
C9—C8—C7—C6	−0.1 (4)	C12—C11—C16—C15	−0.1 (4)
C11—C8—C7—C6	178.2 (3)	C8—C11—C16—C15	179.4 (3)
C13—C14—C17—N3	108.2 (3)	C1—N1—C2—N2	0.6 (4)
C15—C14—C17—N3	−72.2 (4)	C3—N2—C2—N1	−0.7 (3)
O7—Mo3—O6—Mo4	78.17 (8)	C4—N2—C2—N1	179.3 (2)
O8—Mo3—O6—Mo4	−172.77 (6)	C2—N2—C4—C5	−78.1 (3)
O9—Mo3—O6—Mo4	−54.32 (11)	C3—N2—C4—C5	101.9 (3)
O3—Mo3—O6—Mo4	−17.26 (5)	C11—C16—C15—C14	0.3 (5)
O9^i^—Mo3—O6—Mo4	−99.52 (6)	C13—C14—C15—C16	−0.3 (4)
O7—Mo3—O6—Mo2^i^	−178.32 (7)	C17—C14—C15—C16	−180.0 (3)
O8—Mo3—O6—Mo2^i^	−69.27 (7)	N3—C18—N4—C19	0.6 (3)
O9—Mo3—O6—Mo2^i^	49.18 (12)	C20—C19—N4—C18	−0.3 (3)
O3—Mo3—O6—Mo2^i^	86.24 (6)	C2—N2—C3—C1	0.5 (4)
O9^i^—Mo3—O6—Mo2^i^	3.98 (5)	C4—N2—C3—C1	−179.4 (3)
O5—Mo4—O6—Mo3	−75.45 (8)	C7—C6—C5—C10	−0.4 (5)
O4—Mo4—O6—Mo3	85.95 (15)	C7—C6—C5—C4	179.3 (3)
O12^ii^—Mo4—O6—Mo3	−172.07 (8)	C9—C10—C5—C6	−0.4 (4)
O3—Mo4—O6—Mo3	19.10 (6)	C9—C10—C5—C4	179.9 (3)
O11^i^—Mo4—O6—Mo3	105.07 (7)	N2—C4—C5—C6	108.8 (3)
O5—Mo4—O6—Mo2^i^	173.01 (6)	N2—C4—C5—C10	−71.5 (3)
O4—Mo4—O6—Mo2^i^	−25.59 (16)	C11—C12—C13—C14	0.0 (4)
O12^ii^—Mo4—O6—Mo2^i^	76.39 (6)	C15—C14—C13—C12	0.2 (4)
O3—Mo4—O6—Mo2^i^	−92.44 (6)	C17—C14—C13—C12	179.9 (2)
O11^i^—Mo4—O6—Mo2^i^	−6.47 (4)	C5—C10—C9—C8	0.9 (4)
C7—C8—C11—C12	−31.8 (4)	C7—C8—C9—C10	−0.7 (4)
C9—C8—C11—C12	146.5 (3)	C11—C8—C9—C10	−179.0 (2)
C7—C8—C11—C16	148.8 (3)	N2—C3—C1—N1	−0.1 (4)
C9—C8—C11—C16	−32.9 (4)	C2—N1—C1—C3	−0.3 (4)

Symmetry codes: (i) −*x*+1, −*y*+1, −*z*+1; (ii) *x*+1, *y*, *z*; (iii) *x*−1, *y*, *z*.

### Hydrogen-bond geometry (Å, °)

**Table:**

*D*—H···*A*	*D*—H	H···*A*	*D*···*A*	*D*—H···*A*
N1—H1N···O4^iv^	0.92 (3)	1.96 (3)	2.854 (4)	165 (3)
N4—H4N···O13^v^	0.92 (3)	1.77 (2)	2.658 (3)	163 (3)

Symmetry codes: (iv) *x*−1/2, −*y*+3/2, *z*+1/2; (v) −*x*+1/2, *y*−1/2, −*z*+3/2.
